# Phytoestrogens as Biomarkers of Plant Raw Materials Used for Fish Feed Production

**DOI:** 10.3390/molecules28083623

**Published:** 2023-04-21

**Authors:** Dionysios T. Pavlopoulos, Eleni D. Myrtsi, Paschalitsa Tryfinopoulou, Vasilios Iliopoulos, Sofia D. Koulocheri, Serkos A. Haroutounian

**Affiliations:** 1 Laboratory of Nutritional Physiology and Feeding, Department of Animal Science, School of Animal Biosciences, Agricultural University of Athens, Iera Odos 75, 11855 Athens, Greece; 2 Laboratory of Microbiology and Biotechnology of Foods, Department of Food Science and Human Nutrition, School of Food and Nutritional Sciences, Agricultural University of Athens, Iera Odos 75, 11855 Athens, Greece

**Keywords:** phytoestrogens, fish feed, soya, rapeseed, sunflower, wheat, isoflavones, flavones, phenolic acids, LC-MS/MS

## Abstract

The intensive use of plant materials as a sustainable alternative for fish feed production, combined with their phytochemical content, which affects the growth and production characteristics of farmed fishes, necessitates their monitoring for the presence of raw materials of plant origin. This study reported herein concerns the development, validation and application of a workflow using high-performance liquid chromatography combined with tandem mass spectrometry (LC-MS/MS) for the quantification of 67 natural phytoestrogens in plant-derived raw materials that were used to produce fish feeds. Specifically, we verified the presence of 8 phytoestrogens in rapeseed meal samples, 20 in soybean meal samples, 12 in sunflower meal samples and only 1 in wheat meal samples in quantities enabling their efficient incorporation into clusters. Among the various constituents, the soybean phytoestrogens daidzein, genistein, daidzin, glycitin, apigenin, calycosin and coumestrol, as well as the sunflower neochlorogenic, caffeic and chlorogenic phenolic acids, displayed the highest correlations with their origin descriptions. A hierarchical cluster analysis of the studied samples, based on their phytoestrogen contents, led to the efficient clustering of raw materials. The accuracy and efficiency of this clustering were tested through the incorporation of additional samples of soybean meal, wheat meal and maize meal, which verified the utilization of the phytoestrogen content as a valuable biomarker for the discrimination of raw materials used for fish feed production.

## 1. Introduction

The increase in the growth rate of the global population highlights the importance of food sector sustainability to avoid resource depletion and simultaneously maintain the Earth’s ecological balance. Among the various food source industries, fishery and aquaculture are considered to be of great importance, with an annual production volume of 214 million tons in 2020 [1]. Concomitantly, the demand for protein and fat ingredients added to aquaculture feeds has also increased, leading to a demand for the production of large quantities of fishmeal and fish oil [2]. Since both are available in limited quantities, the use of plant materials as a sustainable alternative source of proteins and the lipids contained in fish diets have become essential for semi-intensive or intensified aquacultures [3,4]. In this context, the seeds and flours of various plants have been included among the key widely used protein-rich raw materials, with the most prominent being soybean meal, which has high protein and amino acid contents, a low price and wide availability [5,6,7]. This is alongside rapeseed and sunflower meals, which are derivatives of protein-rich crops and have found a wide range of applications in animal feeding [8]. Moreover, vegetable oils can be used as replacements for fish oils, provided that the balance between essential fatty acids and fish oil supplements is maintained. The applicability of the aforementioned plant materials is enhanced by their wide availability [7,9] in contrast to other plant-based raw materials that are not sufficiently exploited as fish feeds.

**Figure 1:**
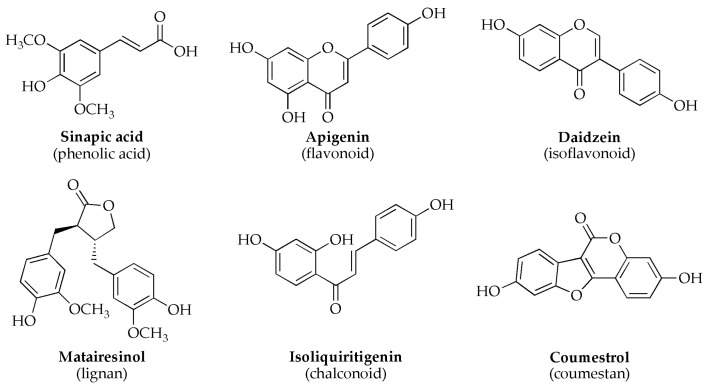
Chemical structures representative of phytoestrogens.

All these protein-rich plant materials, especially soybean, also act as potent sources of non-steroidal secondary metabolites known as phytoestrogens [10]. Phytoestrogens are polyphenolic plant-produced natural compounds that are classified based on their structures: phenolic acids, isoflavonoids, flavonoids, stilbenoids, chalconoids, coumestans and lignans [10,11] (Figure 1). Phytoestrogens are synthesized in plants via various enzymatic pathways, mainly as a response to environmental stress or disease [8]. These molecules are mainly known as protectors of plants from herbivores [6] and for their beneficial roles in plant growth and preservation [7].

The physiological activity of phytoestrogens is mainly connected to their ability to induce biological responses and mimic or alter the activities of endogenous estrogens. They also display an ability to bind with estrogen receptors (ERs) and act as endocrine disruptors by affecting the affinity and number of ERs [12]. The literature abounds with studies that focus on the effects of phytoestrogens, especially genistein, which is contained in soybean meal, and on the reproductive functions of various animals, including fishes [5,11,13]. The effects of genistein have been studied in channel catfish (*Ictalurus punctatu*) [14], *Cyprinus carpio* [3], goldfish (*Carassius auratus*) [15] and *Pimephales promelas* [16]. Phytoestrogens exhibit estrogenic or anti-estrogenic activity by affecting vitellogenesis through their actions on hormonal receptors and affect circulating estradiol levels in male fishes. Additionally, genistein and other phytoestrogens up-regulate the expression of female secondary sexual characteristics in adult male Japanese medaka (*Oryzias latipes*) [17] and induce sex reversal and feminization in rainbow trout (*Oncorhynchus mykiss*) [18,19], Nile Tilapia (*Oreochromis niloticus*) [20] and African catfish (*Clarias gariepinus*) [14].

The controversial effects of phytoestrogens and their beneficial or harmful impacts on fish growth clearly influence the ratios utilized in fish feed production. Thus, the development and application of a modern method for the simultaneous identification of several phytoestrogen analytes and their quantification are important for the monitoring of this ratio. For this purpose, the utilization of mass spectrometry has enabled the reliable identification of metabolites that have emerged from the wide-ranging utilization of this technique, in combination with liquid chromatography, for the recovery and confirmation of metabolic markers [21,22]. Furthermore, the wide range of secondary metabolites renders plant-originating raw materials amenable to metabolic tracer determination [23], while the use of statistical models can facilitate the determination of the metabolite profiles of plant tissues [24]. Thus, it is possible to obtain information that connects plant varieties and cultivation methods with their nutrient contents [22], thus highlighting the usefulness of statistical models to the processing of phytoestrogen analytical data in order to determine raw material origins and detect possible adulterations [25]. These data are essential for both feed producers and aquaculturists who are interested in the nutritional value and authenticity of fish feeds.

In this study, we report on the exploitation of phytoestrogens according to their presence and distribution in various meal samples of wheat (*Triticum* spp.), soybean (*Glycine max*), rapeseed (*Brassica napus*) and sunflower (*Helianthus annuus*), as well as three types of plant-originating commercial oils, all of which are commonly used as raw materials or ingredients in fish feeds.

## 2. Results and Discussion

### 2.1. Extraction of Phytoestrogens

Metabolite extraction is a key step in the development of analytical methods. The choice of extraction conditions is crucial since the efficiency and reproducibility of the procedure must not be affected by a large number of metabolites detected. Phytoestrogens are present in plant material in free forms, such as glucosides and glucuronides [26]. This diversity necessitates an extraction study in order to achieve greater representativeness in the detection of biomarkers. The optimization of parameters involved in the extraction process was carried out using a soybean meal sample. Soybean meal constituents are the most widely studied material for the extraction of isoflavones, and the respective results highlight acetonitrile to be one the most efficient extraction solvents for obtaining a high yield and for the minimization of matrix interference [27]. In order to determine the optimal extraction conditions, a broad variety of ACN/water ratios (60:40, 70:30, 80:20), temperatures (30, 40, and 60 °C) and extraction times (15, 30, 45, and 60 min) were tested in acidic environments (ACN/water mixture with 1% *v*/*v* HCOOH), along with different extraction conditions (e.g., the use of stirring or thermostatic ultrasound-assisted extraction).

In order to select the most fruitful extraction method, the respective recovery rates were determined for each analyte by fortifying the blank samples with a corresponding volume of the selected analyte standard solutions, consisting of compounds representing all classes of phytoestrogens (phenolic acids: chlorogenic acid; isoflavone aglycones: daidzein, biochanin, genistein, glycitein and formononetin; isoflavone glycosides: daidzin, genistin, ononin and sophoricoside; isoflavone glucuronides: daidzein-7-O-glucuronide and genistein-7-O-glucuronide; flavanones: hesperidin; flavones: luteolin, xanthoxumol; coumestans: coumestrol; and chalcones: phloretin and isoliquiritigenin) in 700 ppb concentrations. Each spiked sample was extracted and then analyzed using the LC–MS/MS method. The variations in the recovery rates of the phytoestrogens led to the selection of the one with the optimal profile for the optimal extraction method. The best results were obtained with the utilization of an ACN/water mixture of 70:30 containing 1% *v*/*v* HCOOH left at 40 °C in an ultrasonic bath (37 Hz) for 30 min. In the present extraction study, we further optimized the extraction procedure yield by applying the QuEChERS method [28,29,30], which refers to the utilization of a QuEChERS salt mixture (MgSO_4_/NaCl, 4:1, with shaking for 15 min) to the separation of organic and aqueous phases [31]. This method proved to be effective and led to the extraction of a wide variety of phytoestrogen classes, although their diversity was an obstacle to their recovery.

For the oil samples, the optimization of the phytoestrogens’ extraction parameters was performed using soy oil, which is extensively used in animal and fish feeds [32]. For this purpose, a short comparative study was carried out using the previously applied solvent mixture and changing the extraction parameters, such as the time (30 min to 24 h) and temperature (a room temperature up to 40 °C to avoid glycoside degradation). Thus, extraction for 30 min at room temperature was determined to be the most efficient method since similar phytoestrogen contents were detected from 30 min up to 24 h of extraction. At the end of this process, the C18 sorbent was added to reduce the matrix effect by removing non-polar interferences such as lipids [33].

### 2.2. Quantification and Analytical Method Validation

A run time of 35 min was adequate for the separation of seven phenolic acids (caffeic acid, chlorogenic acid, gallic acid, neochlorogenic acid, p-coumaric acid, protocatechuic acid, sinapic acid), 30 flavonoids (apigenin, catechin, diosmetin, diosmin, epicatechin, epigallocatechin, epigallocatechin gallate, eriodyctiol, gallocatechin, hesperidin, hesperetin, isoquercetin, isorhamnetin, kaempferol, liquiritigenin, liquiritin, luteolin, luteolin-4′-O-glucoside, myricetin, pelargonidin, pelargonin, procyanidin B1, procyanidin B2, quercetagetin, quercetagetin-7-O-glucoside, quercetin, quercitrin, rhamnetin, rutin, and taxifolin), 19 isoflavonoids (3′,4′,7-trihydroxyisoflavone, 4′,6,7-trihydroxyisoflavone, biochanin A, calycosin, calycosin-7-O-d-glycoside, daidzein, daidzein-7-O-glucuronide, daidzin, equol, formononetin, genistein, genistein-7-O-glucuronide, genistin, glycitein, glycitin, ononin, puerarin, sissotrin, and sophoricoside), four chalconoids (isoliquiritigenin, phloretin, phloridzin, and xanthoxumol), three lignans (lariciresinol, matairesinol, and secoisolariciresinol, one coumestan (coumestrol), one phenylethanoil (hydroxytyrosol) and two stilbenoids (polydatin, and resveratrol) following the method developed by Myrtsi et al. [34].

The LOD and LOQ ranged from 1.8 to 120.5 ng/mL and 5.5 to 365.1 ng/mL, respectively. The equol displayed a LOD of 1681.4 ng/mL and LOQ of 5095.2 ng/mL. The accuracy of the recovery of the spiked compounds and the intra-day and intermediate precision were in accordance with the values reported for LC-MS/MS by Myrtsi et al. [34]. The validation results indicate that the analytical method was reliable for metabolomic analyses.

### 2.3. Phytoestrogens in Raw Material Extracts

#### 2.3.1. Phytoestrogen Content

The results of the assessments to determine the presence and quantitation of individual phytoestrogens in the raw material extracts are depicted in Supplementary Table S2, and indicative chromatograms are presented in Supplementary Figure S3. The findings shown herein represent the detailed fingerprinting of 41 phytoestrogens contained in soy, rapeseed, wheat and sunflower meal and include the relevant compounds. Among the detected phytoestrogens, six were phenolic acid derivatives, twelve were isoflavones, seventeen were flavones, three were chalcones, and three were other classes of compounds. Overall, their presence in different concentrations observed over a wide range in the raw materials of fish feeds was indicative of their diversified contents in each material.

A recent study concerning the determination of soybean and rapeseed oil phytoestrogen metabolites identified several markers, including isoliquiritigenin, genistin, formononetin, daidzein, liquiritigenin, genistein, coumestrol, glycitein, biochanin A, daidzin, naringenin, glycitin and sinensetin, in soybean oil, as well as sinapic acid, bergapten, imperatorin, psoralen and kaempherol in rapeseed oil, respectively [35]. In our study, we analyzed commercially available soybean (4), sunflower (4) and rapeseed (4) oils. Our results are in line with the limited amounts of phytoestrogens found in similar samples in the literature surveys. Daidzein (not detected −0.72 μg/g), genistein (not detected −2.1 μg/g) and traces of matairesinol were the only phytoestrogens detected in crude soybean oil. Similar results were obtained for the rapeseed oil extract, in which only sinapic acid (161–251 μg/g) was detected, while neochlorogenic acid (not detected −0.20 μg/g) was the sole phytoestrogen found in sunflower oil. These results can be explained by considering the low solubility of phytoestrogens in non-polar oil extracts since the processes of protein removal, filtration, winterization and application at high temperatures decisively affected their contents in the respective oils [36]. This scarce presence of phytoestrogens in commercial oils prompted us to avoid their incorporation into statistical analysis.

#### 2.3.2. Overview of the Presence of Phytoestrogens in Plant-Based Meals

**Figure 2:**
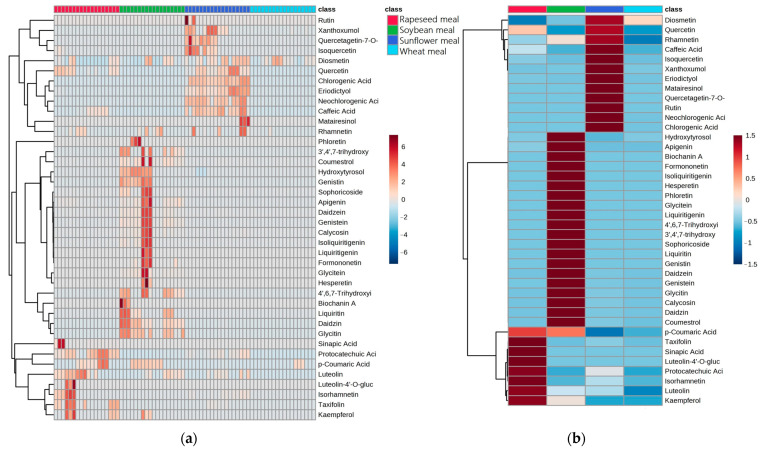
Heatmap of 40 significant phytoestrogens detected in soya, sunflower, rapeseed and wheat meal samples. Original data shown in a heat map (**a**) and gathered with the group averages (**b**), with automatically generated scaled intensity bars.

Based on the results of non-parametric ANOVA determination, a sum of 40 significant metabolites (*p* < 0.005) was detected (Supplementary File, Figure S2). Indeed, the results displayed on the heatmap show that the identified features had significantly variable levels between the study’s raw materials of soya, sunflower, rapeseed and wheat meal, as shown in Figure 2.

The study of the soybean meal samples indicated that isoflavones comprised the most abundant group of compounds, displaying the highest concentrations and number of detected molecules since daidzein, genistein and glycitein were found in all the samples. These findings are consistent with previous reports in the literature [37,38,39,40]. In parallel, the presence of the main glycosides was revealed, while molecules of 3′,4′,7-trihydroxyisoflavone, 4′,6,7-trihydroxyisoflavone and calycosin were detected in most of the samples. Molecules of formononetin and sophoricoside were found in only one sample, and liquiritigenin traces were detected in all the samples [41]. It is also reported in the literature that soya samples contain flavonoids [40,42]. Herein, only apigenin, pelargonidin and diosmetin were revealed as being present in the studied samples, while molecules of luteolin, liquiritin kaempferol and isoliquiritigenin were detected in a limited number of samples. Phenolic acids comprise an additional class of phytoestrogens that are frequently detected in soya extracts [23,43,44]. Among the four phenolic acids detected herein, neochlorogenic acid was the most abundant, while protocatechuic, chlorogenic and p-coumaric acids were detected in half of the samples. Our results are in accordance with Hutabarat et al. [45], who noted the presence of coumestrol in soya samples.

In total, the presence of 21 metabolites was revealed in the rapeseed samples. Sinapic acid was the most abundant, while neochlorogenic, protocatechuic, caffeic and chlorogenic acids, representing phenolic acids, were detected in all the samples, in line with the literature [46,47,48]. Molecules of luteolin, quercetin and apigenin were found in significant quantities, while the metabolites taxifolin, diosmetin, kaempferol and isorhamnetin occurred in a small number of samples and in significantly lower concentrations. Finally, traces of daidzin, genistein, daidzein, glycitin, rhamnetin and coumestrol were detected in only one sample.

In the sunflower samples, molecules of neochlorogenic and chlorogenic acids were observed in the highest concentrations, with caffeic acid and quercetin being the next most abundant molecules, in agreement with the literature reports [49,50,51,52,53]. In addition, molecules of eriodyctiol, diosmetin, rhamnetin, isorhamnetin, protocatechuic acid and quercetagetin-7-O-glucoside were also detected, confirming the findings of Abdalla et al. [54], while traces of daidzin, genistein, daidzein, glycitin, rhamnetin and coumestrol were detected in only one sample.

As anticipated, the number of wheat-derived metabolites was significantly lower [55,56], with a 10–20% rate of occurrence for phenolic acids, including neochlorogenic, chlorogenic, caffeic and protocatechuic acids. Only diosmetin displayed an occurrence rate that reached the 80% mark.

The data separating the different matrices of phytoestrogens that were determined to be characteristics of every class of raw materials are shown in Figure 2b. The rapeseed meal samples were mainly composed of kaempferol, luteolin, isorhamnetin, protocatechuic acid, luteolin-4-O-glucose, sinapic acid, taxifolin and p-coumaric acid. Correspondingly, the soybean meal samples were characterized by coumestrol, daidzin, calycosin, daidzein, genistein, genistin, liquiritin, sophoricoside, 3′,4′,7-trihydroxyisoflavone, 4′,6,7-trihydroxyisoflavone, liquiritigenin, glycitein, phloretin, hesperetin, isoliquiritigenin, formonetin, biochanin A, apigenin and hydroxytyrosol. Neochlorogenic acid, chlorogenic acid, rutin, quercetagetin-7-O-glucoside, matairesinol, eriodictyol, xanthoxumol, isoquercetin, caffeic acid, quercetin and diosmetin were present in the sunflower meal samples. Finally, diosmetin was observed only in the wheat meal samples.

**Figure 3:**
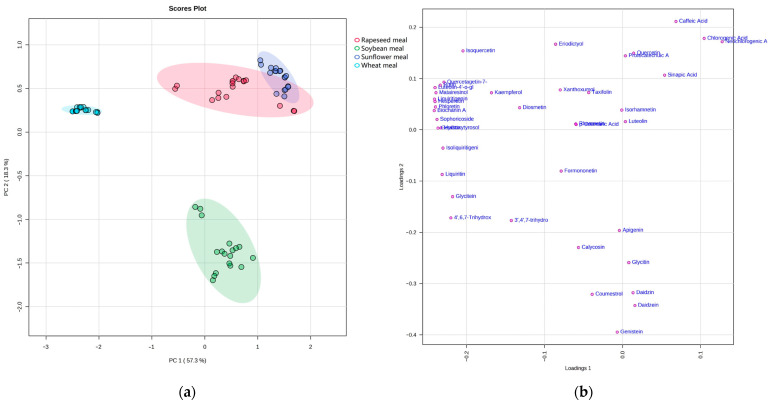
Score plot of principal component analysis (PCA) of (**a**) LC-MS-based normalized data and (**b**) Loading plot of phytoestrogens.

Principal component analysis (PCA) was performed with the normalized data. The respective results are depicted in Figure 3, which provides the clustering of the different raw materials and depicts the distribution relationships between the phytoestrogens in the studied samples.

In the PCA diagram, component 1 and component 2 represent 57.3% and 18.3% of the variance (Figure 3), respectively, indicating that there is a significant difference between the raw materials. This analysis also shows a distinct separation between the soybean and wheat and an overlap between the sunflower and rapeseed samples, presumably due to contamination.

**Figure 4:**
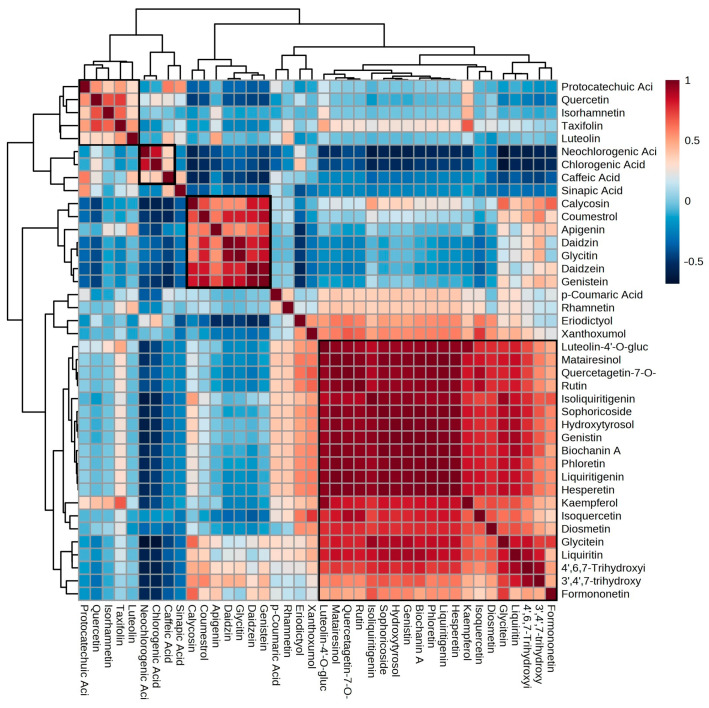
Correlation heatmap depicting the relationships between the phytoestrogens.

Correlation analysis is a statistical method that is often used to delineate the relationships between metabolites in a biological system. In the present study, it was performed to obtain information about the relationships between the 40 phytoestrogens studied herein (Figure 4). The soybean metabolite family consisted of daidzein, genistein, daidzin, glycitin, apigenin, calycosin and coumestrol, which displayed very high correlations and could possibly be used as soybean biomarkers. The phenolic acids neochlorogenic, caffeic and chlorogenic acids detected in the sunflower samples were also clustered, showing very strong relationships, especially considering that between the neochlorogenic and chlorogenic acids. In the case of rapeseed, the most abundant acid, sinapic acid, was distinguished from the other metabolites. It showed a significant correlation with protocatechuic acid and weak correlations with luteolin, taxifolin and isorhamnetin, which were grouped with quercetin. Sinapic acid also displayed a correlation with caffeic acid. Finally, many of the metabolites were clustered together and showed strong correlations, but they could not be assigned to a single raw material.

**Figure 5:**
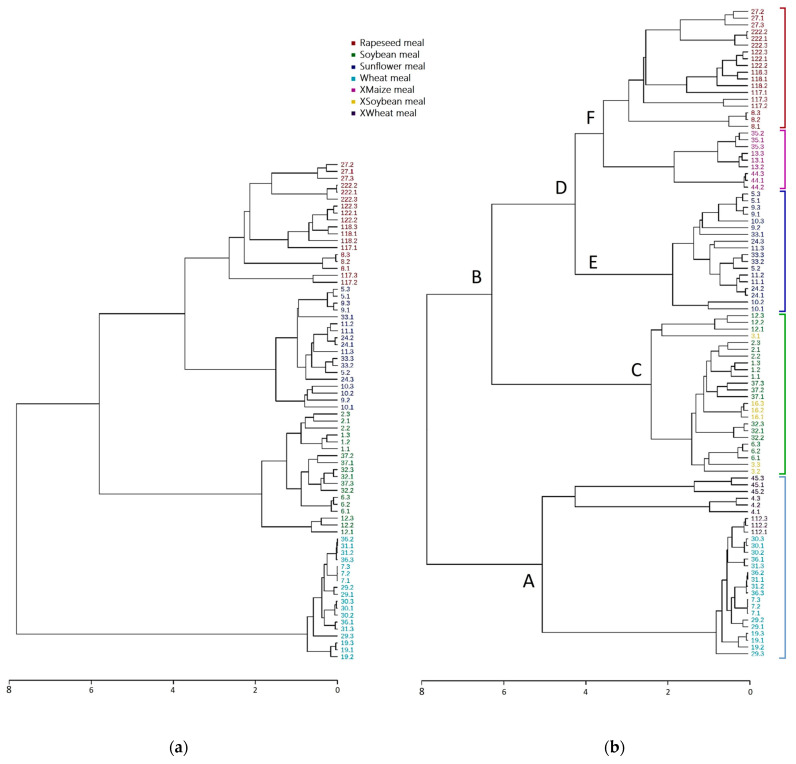
Hierarchical clustering analysis of the studied samples (**a**), with the presence of the additional (**b**) samples included in the present study. Samples marked with X before their names indicate additional meal samples of soya, wheat and maize.

Finally, hierarchical clustering analysis was performed to confirm the raw material sample grouping based on the phytoestrogen contents, as shown in Figure 5a. The soya, sunflower, wheat, and rapeseed samples were clustered with more than 80% and 75% similarity, respectively, forming distinctively separate groups. When additional samples of soybean, wheat and maize meals were added to the analysis, the cluster analysis provided the dendrogram shown in Figure 5b. Following the grouping of the first dendrogram, we obtained a first-level separation of the raw material samples into two distinct and main clusters, A and B. Cluster A consisted of wheat samples displaying the characteristics of low concentrations and a wide diversity of phytoestrogens. The additional samples of the wheat meal were grouped in the wheat meal cluster A, and only one was subclustered with the wheat samples. Cluster B was divided into two subclusters, C and D. Subcluster C corresponded to soymeal, with the additional soybean meal sample also joining this soya subcluster. Correspondingly, the samples in subgroup D were also divided into two subclusters, E and F. Subcluster E consisted of sunflower samples, while subcluster F was divided into rapeseed and maize subclusters.

The distinct clustering of the raw materials, the incorporation of their meals, and the finding that the maize sample was not grouped with any of the soybean, sunflower, rapeseed and wheat meals but was grouped alone were indicative of the accuracy and practicability of this method and the usefulness of the phytoestrogen content as a biomarker for discriminating between the raw materials used to produce fish feed.

## 3. Materials and Methods

### 3.1. Sample Preparation

The 26 plant-derived samples of the fish feed raw materials studied herein were obtained from various outlets in Greece and included six (6) samples of soybean, sunflower, rapeseed and wheat meal, respectively, and additional samples of soymeal (2), wheatmeal (3), maize meal (3), soya oil (4), sunflower oil (4) and rapeseed oil (4). The samples were stored at room temperature, except for the oils, which were lyophilized before storage. Each sample’s analysis was performed in triplicate using the method optimized in this study.

### 3.2. Chemicals and Reagents

The phytoestrogen standards used in the analytical method were purchased from ExtraSynthese (Lyon, France), except for puerarin, equol and calycosin, which were obtained from TCI (Tokyo Chemical Industry; Tokyo, Japan), and calycosin-7-O-β-D-glucoside, lariciresinol, matairesinol and secoisolariciresinol, which were obtained from Biosynth Carbosynth (Compton, UK). The molecule of 2-(4-chlorophenyl) malonaldehyde, used as the internal standard (I.S.), was purchased from Sigma-Aldrich (St. Louis, MO, USA). Acetonitrile (ACN) and water (LC-MS grade) were purchased from J.T. Baker (Phillipsburg, NJ, USA), while the formic acid (LC-MS grade), dimethyl sulfoxide (DMSO) and analytical-grade solvents used for the sample extractions (acetonitrile, methanol and acetic acid) were obtained from Fischer Chemicals (Hampton, NH, USA). Magnesium sulphate anhydrous (MgSO_4_) and sodium chloride (analytical grade) were obtained from Chem-Lab (Zedelgem, Belgium), while LiChroprep RP-C18 (40–63 µm) was provided by Merck (Rahway, NJ, USA).

### 3.3. Extraction of Phytoestrogens from Raw Materials

#### 3.3.1. Phytoestrogen Extraction from Plant Raw Materials of Meals

A total of 1 g of each meal sample was weighed in a 25 mL propylene centrifuge tube, the required amount of the respective I.S. solution was added, and the sample was allowed to dry under an N_2_ stream. Then, 10 mL of solvent (ACN/water 70:30, 1% *v*/*v* HCOOH) was added, and the tube was placed in a thermostatic ultrasonic water bath at 40 °C for 30 min. Subsequently, 1.250 g of the original QuEChERS extraction salts (MgSO_4_: NaCl, 4:1) was added into the centrifuge tube, and the mixture was stirred for 15 min. The sample was centrifuged at room temperature for 5 min at 4000 rpm, and the supernatant was separated and filtrated through a 0.22 μm PVDF syringe filter before it was immediately analyzed.

#### 3.3.2. Phytoestrogen Extraction from Oils

A total of 10 mL of each oil sample was placed in 15 mL of solvent (ACN/water 70:30, 1% *v*/*v* HCOOH) in a round-bottomed glass flask and stirred at room temperature for 30 min. After being left to settle, the two phases were separated, and the aqueous layer was removed. Then, 12 mg of the C18 sorbent was added into the organic phase, which was shaken in a vortex for 1 min and centrifuged as described above. The supernatant was separated and filtrated through a 0.22 μm PVDF syringe filter and injected into the HPLC-MS/MS system.

### 3.4. LC–MS/MS Analysis

#### 3.4.1. Preparation of Standard Solutions

Each analyte standard was diluted in DMSO or methanol for the preparation of the respective stock solution (2000 to 6000 μg/mL). Quantification was achieved by diluting the stock standard solutions of the target analyte in order to construct the calibration curve of each analyte at 9 different concentrations (10, 20, 50, 100, 300, 500, 800, 1000 and 1400 ng/mL). The internal standard solution was used at a concentration of 200 ng/mL. All standard solutions were stored in the dark at −20 °C.

#### 3.4.2. Liquid Chromatography/Mass Spectrometry

The samples were analyzed using an Accela Ultra-High-Performance Liquid Chromatography system coupled with a TSQ Quantum Access triple-quadrupole mass spectrometer utilizing LCquan 2.7.0.20 software (Thermo Fisher Scientific, Inc., Waltham, MA, USA) and equipped with an autosampler.

For the separation of the phytoestrogens, a Fortis C18 column with a 150 × 2.1 mm i.d. and 3 μm particle size (Fortis Technologies Ltd., Neston, Cheshire, UK) was used, in combination with an AF C18 guard column from the same provider, with a 10 × 2.0 mm i.d. and 3 μm particle size.

Gradient elution was applied using mobile phase A, comprising water and formic acid (0.1%), and phase B comprising acetonitrile (ACN). The injection volume of the samples was set at 10 μL, and the mobile flow rate was 0.28 mL/min. The conditions of the gradient elution used to re-equilibrate the column between injections were set as follows: 0.0–2.0 min, 20% B; 2.0–25.0 min, 20% to 51% B; 25.0–30.0 min, 51% to 70% B; 30.0–30.1 min, 20% B; and 30.1–35.0 min, 20% B. The column temperature was maintained at 32 °C, and the tray temperature was maintained at 25 °C throughout the analysis.

MS/MS determination was performed using ElectroSpray Ionization (ESI) in both the positive and negative ion modes and the Selected Reaction Monitoring mode (SRM). The direct injection procedure in the full-scan mode (mass range: 100–1000) was used to determine the molecular ion transitions of the target analytes and the corresponding collision energies. The capillary temperature was adjusted to 300 °C. A nitrogen generator (Peak Scientific, Inchinnan, UK) provided nitrogen, which was used as a sheath and auxiliary gas. The initial gas pressures were set at 35 and 10 Arb, respectively. The collision pressure of the argon gas (99.9999%) was regulated at 1.5 mTorr, and the spray voltage was set to 3.5 kV in both polarities (positive/negative).

### 3.5. Quantification of Phytoestrogens and Method Validation

The linearity of the calibration curves described above was determined as a regression coefficient ≥0.998, with detected residues of ≤20%. The analytical method’s validation was performed in accordance with a method recently reported by Myrtsi et al. [34], i.e., by determining the respective limit of detection (LOD), the limit of quantification (LOQ), precision (expressed as the relative standard deviation), the %RSD of intra-day repeatability and inter-day intermediate precision of three analyses (*n* = 3), the recovery value of each analyte and the matrix effect. The equations, regression coefficient, LOD and LOQ are depicted in Table S1 of the Supplementary Materials.

### 3.6. Data Pre-Treatment and Statistics

The generated table consisted of concentrations that were submitted to the web-based MetaboAnalyst 5.0 platform. Prior to statistical analysis, all data values were preprocessed through sum normalization, square root transformation and range scaling. This study aimed to analyze the significance of the normalized data and identify interesting features; therefore, further data analysis of variance, principal component analysis, and correlation and hierarchical clustering were carried out using the Pearson distance and average linkage.

## 4. Conclusions

Four raw materials of plant origin that are used for the production of fish feed were investigated for the determination of their phytoestrogen contents. The results highlight that several phytoestrogens display strong correlations and may be useful as biomarkers for the efficient detection of soybean and sunflower in feedstocks. The described screening method for these biomarkers may be generally applicable to other raw materials. The observed distinct groupings of crude raw materials, in combination with the incorporation of their flours and the distinct clustering of corn meal, highlight the utility of this method and the possible advantages of its use for monitoring fish feed, in animal feed production and, potentially, in the production of foodstuffs containing industrial raw materials.

## Data Availability

The data are contained within the article.

## Supplementary Materials

The following supporting information can be downloaded at: https://www.mdpi.com/article/10.3390/molecules28083623/s1, Table S1: Transition, collision energy, polarity, retention time (RT), calculation of equations, determination coefficients, LOD and LOQ for each phytoestrogen standard; Table S2: Phytoestrogen concentration ranges of soymeal, sunflower meal, rapeseed meal and wheat meal; Figure S1: Concentration distributions before and after normalization based on the kernel density. Similarly, the bottom plots graphically summarize the concentrations of individual compounds; Figure S2: Results of the analysis of variance (ANOVA) of phytoestrogens, ranked by their *p* values (*p* < 0.05); Figure S3: Chromatograms of phytoestrogens extracted from samples: (a) soybean, (b) rapeseed, (c) sunflower and (d) wheat meals.

## Sample Availability

Samples of the compounds are not available from the authors.
