# Supplementary material for: Phytoestrogens as Biomarkers of Plant Raw Materials Used for Fish Feed Production
Source: Molecules. 2023 Apr 21;28(8):3623. doi: 10.3390/molecules28083623 (PMC10144496; doi:10.3390/molecules28083623)
Supplement: Supplementary file 1 [file molecules-28-03623-s001.zip › molecules-2314620-supplementary.pdf]

# Phytoestrogens as biomarkers of plant raw materials used for fish feed production

**Dionysios T. Pavlopoulos [1], Eleni D. Myrtsi[1], PaschalitsaTryfinopoulou[2], Vasilios Iliopoulos[1], Sofia D. Koulocheri[1], * and Serkos A. Haroutounian[1]**

# Supplementary Materials

**Table S1.** Transition, collision energy, polarity, retention time (RT), calculation of equations, determination coefficients, LOD and LOQ for each phytoestrogen standard

**Table S2**. Phytoestrogen concentration ranges of soymeal, sunflower meal, rapeseed meal and wheat meal.

**Figure S1**. Concentration distributions before and after normalization based on the kernel density. Similarly, the bottom plots graphically summarize the concentrations of individual compounds.

**Figure S2**. Results of the analysis of variance (ANOVA) of phytoestrogens, ranked by their P values (P<0.05)

**Figure S3.** Chromatograms of phytoestrogens extracted from samples of soybean (a), rapeseed (b), sunflower (c) and wheat (d) meals.

**Table S1.** Transition, collision energy, polarity, retention time (RT), calculation of equations, determination coefficients, LOD and LOQ for each phytoestrogen standard.

| | Compound | Parent Mass | Product Mass | Colision Energy (eV) | Polarity | RT (min) | LOD (ng/mL) | LOQ (ng/mL) | Equation | $R^2$ |
|---|---|---|---|---|---|---|---|---|---|---|
| | ***Phenolic acids*** | | | | | | | | | |
| 1 | Caffeic Acid | 180.0 | 135.9 | 19 | (-) | 3.89 | 51.4 | 155.8 | Y = 0.00205272+0.800781*X | 0.9997 |
| 2 | Chlorogenic Acid | 354.2 | 191.7 | 22 | (-) | 2.36 | 44.6 | 135.1 | Y = 0.00242681+0.0435718*X | 0.9988 |
| 3 | Gallic Acid | 170.1 | 126.0 | 18 | (-) | 2.05 | 73.1 | 221.5 | Y = -0.000734999+0.247545*X | 0.9998 |
| 4 | Neochlorogenic Acid | 354.2 | 190.9 | 21 | (-) | 2.04 | 12.6 | 38.2 | Y = 0.00126487+0.332059*X | 0.9992 |
| 5 | p-Coumaric Acid | 164.0 | 120.0 | 18 | (-) | 6.16 | 46.0 | 139.5 | Y = 0.00445059+0.384097*X | 0.9998 |
| 6 | Protocatechuic Acid | 154.1 | 110.0 | 18 | (-) | 2.37 | 16.2 | 49.1 | Y = 0.00114193+0.336255*X | 0.9996 |
| 7 | Sinapic Acid | 224.1 | 208.9 | 16 | (-) | 6.78 | 54.9 | 166.2 | Y = 0.00292367+0.00171603*X | 0.9999 |
| | ***Flavonoids*** | | | | | | | | | |
| 8 | Apigenin | 270.0 | 118.1 | 42 | (-) | 16.98 | 8.7 | 26.4 | Y = -0.00105394+1.4346*X | 0.9992 |
| 9 | Catechin | 290.1 | 204.1 | 21 | (-) | 2.9 | 75.5 | 228.9 | Y = 0.0467805+0.934157*X | 0.9996 |
| 10 | Diosmetin | 300.3 | 285.0 | 20 | (-) | 17.67 | 53.9 | 163.3 | Y = 0.000344824+0.00220693*X | 1.0000 |
| 11 | Diosmin | 608.4 | 607.6<br>284.1 | 13 | (-) | 8.1 | 12.5 | 37.7 | Y = -0.000727451+0.146644*X | 0.9983 |
| 12 | Epicatechin | 290.2 | 246.3 | 18 | (-) | 3.57 | 18.9 | 57.3 | Y = 0.0105637+0.0205232*X | 0.9993 |
| 13 | Epigallocatechin | 306.2 | 125.0 | 29 | (-) | 2.47 | 39.2 | 118.9 | Y = 0.00338522+0.0195243*X | 0.9996 |
| 14 | Epigallocatechin Gallate | 458.2 | 168.8 | 21 | (-) | 3.44 | 30.7 | 93.1 | Y = -0.0193777+0.909754*X | 0.9993 |
| 15 | Eriodictyol | 288.2 | 151.2 | 17 | (-) | 12.67 | 23.6 | 71.6 | Y = -0.00580681+0.335251*X | 0.9996 |
| 16 | Gallocatechin | 306.1 | 125.0 | 24 | (-) | 2.23 | 109.5 | 331.8 | Y = 0.0110903+0.0536722*X | 0.9993 |
| 17 | Herperidin | 610.1 | 301.7 | 26 | (-) | 7.78 | 27.2 | 82.3 | Y = -0.0393733+3.85778*X | 0.9991 |
| 18 | Hesperetin | 302.1 | 286.9 | 20 | (-) | 17.37 | 13.8 | 42.0 | Y = 0.000690733+0.189778*X | 0.9991 |
| 19 | Isoquercetin | 464.0 | 300.7 | 27 | (-) | 6.42 | 46.2 | 140.0 | Y = -8.85249e-005+0.0177665*X | 0.9996 |

| | Compound | Parent Mass | Product Mass | Colision Energy (eV) | Polarity | RT (min) | LOD (ng/mL) | LOQ (ng/mL) | Equation | $R^2$ |
|---|---|---|---|---|---|---|---|---|---|---|
| 20 | Isorhamnetin | 316.1 | 301.0 | 24 | (-) | 18.08 | 47.5 | 144.0 | Y = 3.04779e-005+0.0167596*X | 0.9998 |
| 21 | Kaempferol | 286.1 | 240.0 | 31 | (-) | 17.46 | 15.0 | 45.5 | Y = 0.00622424+0.0165355*X | 0.9999 |
| 22 | Liquiritigenin | 256.2 | 135.0 | 18 | (-) | 12.64 | 77.4 | 234.7 | Y = 0.0106288+0.760192*X | 0.9989 |
| 23 | Liquiritin | 418.2 | 256.0 | 24 | (-) | 5.96 | 26.3 | 79.6 | Y = -0.000555141+0.784532*X | 0.9997 |
| 24 | Luteolin | 286.1 | 133.0 | 36 | (-) | 13.74 | 20.6 | 62.3 | Y = 0.000398407+1.0949*X | 0.9994 |
| 25 | Luteolin-4'-o-glucoside | 448.3 | 284.9 | 21 | (-) | 8.62 | 29.8 | 90.3 | Y = -0.000145195+0.0271768*X | 0.9992 |
| 26 | Myricetin | 318.1 | 150.8 | 26 | (-) | 9.85 | 67.1 | 203.2 | Y = -0.00126106+0.200528*X | 0.9993 |
| 27 | Pelargonidin | 271.0 | 121.0 | 33 | (+) | 5.20 | 23.3 | 70.5 | Y = 1.18774+4.54226*X | 0.9994 |
| 28 | Pelargonin | 595.2 | 270.9 | 33 | (+) | 2.05 | 7.1 | 21.4 | Y = -1.33029+242.479*X | 0.9995 |
| 29 | Procyanidin B1 | 578.3 | 407.7 | 25 | (-) | 2.3 | 46.9 | 142.2 | Y = -0.0157375+1.99064*X | 0.9997 |
| 30 | Procyanidin B2 | 578.3 | 408.3 | 29 | (-) | 2.83 | 55.0 | 166.8 | Y = -0.000121611+0.0297545*X | 0.9991 |
| 31 | Quercetagetin | 318.1 | 139.1 | 32 | (-) | 8.49 | 61.1 | 185.2 | Y = -0.0126383+0.591688*X | 0.9995 |
| 32 | Quercetagetin-7-o-glucoside | 480.3 | 317.7 | 25 | (-) | 3.91 | 29.8 | 90.2 | Y = -0.0195021+1.5577*X | 0.9996 |
| 33 | Quercetin | 302.1 | 151.0 | 25 | (-) | 13.74 | 17.9 | 54.3 | Y = -0.00174477+0.284061*X | 0.9993 |
| 34 | Quercitrin | 448.2 | 300.7 | 28 | (-) | 8.3 | 38.5 | 116.8 | Y = -0.0597938+6.41218*X | 0.9986 |
| 35 | Rhamnetin | 316.1 | 165.5 | 25 | (-) | 20.58 | 13.3 | 40.3 | Y = -0.000444812+0.557033*X | 0.9992 |
| 36 | Rutin | 610.3 | 300.6 | 36 | (-) | 5.38 | 102.2 | 309.5 | Y = 0.0749463+3.00856*X | 0.9996 |
| 37 | Taxifolin | 304.1 | 286.0 | 15 | (-) | 7.19 | 17.2 | 52.2 | Y = -0.000622447+0.252727*X | 0.9987 |
| | ***Isoflavonoids*** | | | | | | | | | |
| 38 | 3'.4'.7-trihydroxyisoflavone | 270.1 | 242.1 | 24 | (-) | 9.01 | 21.8 | 65.9 | Y = 0.144262+0.669305*X | 0.9998 |
| 39 | 4'.6.7-Trihydroxyisoflavone | 270.1 | 241.0 | 31 | (-) | 9.66 | 1.8 | 5.5 | Y = -0.00907706+1.71037*X | 1.0000 |
| 40 | Biochanin A | 284.1 | 268.8 | 21 | (-) | 25.08 | 11.0 | 33.2 | Y = 0.000563934+3.01299*X | 0.9998 |
| 41 | Calycosin | 284.1 | 268.9 | 21 | (-) | 13.29 | 22.2 | 67.3 | Y = 0.00514076+2.78286*X | 0.9995 |
| 42 | Calycosin-7-O-D-glycoside | 446.3 | 445.6<br>368.0 | 14 | (+) | 446.305 | 21.8 | 66.2 | Y = -6.88702e-005+0.0820548*X | 0.9993 |
| 43 | Daidzein | 254.3 | 253.6 | 15 | (-) | 254.287 | 51.3 | 155.3 | Y = 0.000239129+2.1368*X | 0.9997 |

| | Compound | Parent Mass | Product Mass | Colision Energy (eV) | Polarity | RT (min) | LOD (ng/mL) | LOQ (ng/mL) | Equation | $R^2$ |
|---|---|---|---|---|---|---|---|---|---|---|
| 44 | Daidzein-7-O-glucuronide | 430.3 | 254.0 | 32 | (-) | 4.15 | 52.0 | 157.4 | Y = 3.55407e-006+0.13471*X | 0.9994 |
| 45 | Daidzin | 416.7 | 254.8 | 26 | (+) | 4.02 | 57.1 | 173.0 | Y = 0.000401047+0.0236351*X | 0.9991 |
| 46 | Equol | 242.3 | 121.5 | 17 | (-) | 16.38 | 1681.4 | 5095.2 | Y = -0.000448591+0.000405631*X | 0.9991 |
| 47 | Formononetin | 268.2 | 252.9 | 23 | (-) | 19.69 | 30.5 | 92.4 | Y = 0.00559216+2.84812*X | 0.9989 |
| 48 | Genistein | 270.1 | 133.8 | 37 | (-) | 16.66 | 18.1 | 54.8 | Y = 0.0507112+3.84183*X | 0.9996 |
| 49 | Genistein-7-O-glucuronide | 446.3 | 268.8 | 31 | (-) | 7.32 | 47.1 | 142.7 | Y = -0.00305471+0.563188*X | 0.9993 |
| 50 | Genistin | 432.3 | 268.9 | 32 | (-) | 8.43 | 12.4 | 37.6 | Y = -0.000508358+0.187553*X | 0.9992 |
| 51 | Glycitein | 284.2 | 268.6 | 24 | (-) | 13.42 | 10.7 | 32.5 | Y = 0.000638602+2.78027*X | 0.9994 |
| 52 | Glycitin | 446.8 | 284.8 | 26 | (+) | 5.21 | 70.5 | 213.7 | Y = -4.05352+1140.78*X | 0.9991 |
| 53 | Ononin | 430.7 | 268.8 | 21 | (+) | 10.24 | 20.8 | 62.9 | Y = -6.33165+830.735*X | 0.9993 |
| 54 | Puerarin | 416.2 | 295.6 | 24 | (-) | 2.79 | 47.5 | 144.0 | Y = 0.000211845+0.00598467*X | 0.9989 |
| 55 | Sissotrin | 446.3 | 283.9 | 22 | (-) | 14.06 | 33.8 | 102.3 | Y = -0.000233527+0.0801326*X | 0.9998 |
| 56 | Sophoricoside | 432.3 | 431.6<br>270.1 | 19 | (-) | 8.3 | 20.5 | 62.1 | Y = 0.0606486+6.68509*X | 0.9993 |
| | ***Chalconoids*** | | | | | | | | | |
| 57 | Isoliquiritigenin | 256.2 | 120.0 | 29 | (-) | 19.67 | 13.7 | 41.4 | Y = -0.000899955+1.59136*X | 0.9995 |
| 58 | Phloretin | 274.1 | 167.9 | 19 | (-) | 16.27 | 20.6 | 62.3 | Y = -0.000347951+1.54746*X | 0.9994 |
| 59 | Phloridzin | 436.2 | 273.9 | 19 | (-) | 9.13 | 32.4 | 98.2 | Y = -0.00080977+0.23241*X | 0.9995 |
| 60 | Xanthoxumol | 354.2 | 234.0 | 21 | (-) | 31.82 | 81.4 | 246.6 | Y = -0.00111669+0.00335894*X | 0.9999 |
| | ***Lignans*** | | | | | | | | | |
| 61 | Lariciresinol | 360.2 | 330.0 | 12 | (-) | 9.98 | 42.6 | 129.1 | Y = 0.0036846+0.00281203*X | 0.9999 |
| 62 | Matairesinol | 358.2 | 343.0<br>82.9 | 23 | (-) | 15.88 | 72.2 | 218.9 | Y = 0.00327439+0.0181311*X | 0.9989 |
| 63 | Secoisolariciresinol | 362.3 | 361.5<br>165.8 | 11 | (-) | 9.4 | 98.8 | 299.4 | Y = 0.00211407+0.247818*X | 0.9988 |
| | ***Coumestan*** | | | | | | | | | |
| 64 | Coumestrol | 268.1 | 239.9 | 26 | (-) | 17.23 | 4.0 | 12.1 | Y = 0.0238974+1.11973*X | 0.9995 |

| | Compound | Parent Mass | Product Mass | Colision Energy (eV) | Polarity | RT (min) | LOD (ng/mL) | LOQ (ng/mL) | Equation | $R^2$ |
|---|---|---|---|---|---|---|---|---|---|---|
| | ***Phenylethanoil*** | | | | | | | | | |
| 65 | Hydroxytyrosol | 154.1 | 124.1 | 17 | (-) | 2.25 | 33.8 | 102.3 | Y = 0.000315019+0.114629*X | 0.9999 |
| | ***Stilbenoids*** | | | | | | | | | |
| 66 | Polydatin | 390.2 | 227.7 | 20 | (-) | 5.94 | 39.4 | 119.4 | Y = -0.0178066+4.15442*X | 0.9984 |
| 67 | Resveratrol | 228.0 | 184.1 | 24 | (-) | 11.41 | 120.5 | 365.1 | Y = 0.0146079+0.0367231*X | 0.9984 |

ng/mL: ng of std/mL of extract

**Table S2.** Phytoestrogen concentration range in soya, sunflower, rapeseed, and wheat meals.

| Compound | SOYA (μg/g) | SUNFLOWER (μg/g) | RAPESEED (μg/g) | WHEAT (μg/g) |
|---|---|---|---|---|
| ***Phenolic acids*** | | | | |
| Caffeic Acid | n.d. | 15.6–34.8 | 0.5–9.4 | n.d. –2.1 |
| Chlorogenic Acid | n.d.–1.11 | 132.1–261.6 | 0.23–2.1 | n.d. –1.2 |
| Neochlorogenic Acid | 0.22–3.24 | 205.3–441.8 | 0.44–2.6 | n.d. –7.8 |
| p-Coumaric Acid | n.d.–1.65 | n.d. –0.101 | n.d.–3.2 | n.d. –1.11 |
| Protocatechuic Acid | n.d.–0.263 | 0.9–3.9 | 1.5–13.5 | n.d.-0.21 |
| Sinapic Acid | n.d. | n.d. | 10.13– >1,000 | n.d. |
| ***Flavonoids*** | | | | n.d. |
| Apigenin | 1.1–18.0 | n.d. | n.d.–0.7 | n.d. |
| Diosmetin | 0.03–0.75 | 0.17–0.66 | n.d.–0.70 | n.d.–0.782 |
| Eriodictyol | n.d. | 0.69–2.8 | n.d.–0.14 | n.d. |
| Hesperetin | n.d. –0.05 | n.d. | n.d. | n.d. |
| Isoquercetin | tr | n.d.–0.08 | n.d. | n.d. |
| Isorhamnetin | n.d. | n.d.–0.262 | n.d.–2.6 | n.d. –0.024 |
| Kaempferol | n.d.–0.6 | n.d. | n.d.–1.7 | n.d. |
| Liquiritigenin | n.d.-0.4 | n.d. | n.d. | n.d. |
| Liquiritin | n.d.–0.121 | n.d. | n.d. | n.d. |
| Luteolin | n.d.–0.31 | n.d.–0.40 | n.d.–0.88 | n.d. |
| Luteolin-4'-O-glucoside | n.d. | n.d. | n.d.–0.04 | n.d. |
| Pelargonidin | n.d. | n.d. | n.d. | n.d. |
| Quercetagetin-7-O-glucoside | n.d. | n.d.–0.2 | n.d. | n.d. |
| Quercetin | n.d. | 3.63–38.5 | 0.6–19.63 | n.d.–0.136 |
| Rhamnetin | n.d.–0.03 | n.d.–0.068 | n.d.–0.009 | n.d.–tr |
| Rutin | n.d. | n.d. –0.03 | n.d. | n.d. |
| Taxifolin | n.d. | n.d. –0.104 | n.d.–1.4 | n.d. |
| ***Isoflavonoids*** | | | | |
| 3'.4'.7-trihydroxyisoflavone | n.d.–2.6 | n.d. | n.d. | n.d.–0.115 |
| 4'.6.7-Trihydroxyisoflavone | n.d.–14.3 | n.d. | n.d. | n.d. |
| Biochanin A | n.d.–0.012 | n.d. | n.d. | n.d. |
| Calycosin | 0.04–3.3 | n.d.–0.047 | n.d. | n.d. |
| Daidzein | 0.63–36.5 | n.d.–0.46 | n.d.–0.05 | n.d. |
| Daidzin | 2.1–11.6 | n.d.-0.34 | n.d.–0.1 | n.d.–0.26 |
| Formononetin | n.d.–0.22 | n.d. | n.d. | n.d. |
| Genistein | 36.3–670.4 | n.d.–4.5 | n.d.–2.27 | n.d. |
| Genistin | n.d.–0.063 | n.d. | n.d. | n.d. |
| Glycitein | 0.06–2.2 | n.d. | n.d. | n.d.–0.9 |

| | | | | |
|---|---|---|---|---|
| Glycitin | 0.44–0.63 | n.d.–0.016 | n.d.–0.003 | n.d.–0.15 |
| Sophoricoside | n.d.–0.110 | n.d. | n.d. | n.d. |
| ***Chalconoids*** | | | | |
| Isoliquiritigenin | n.d.–0.63 | n.d. | n.d. | n.d. |
| Phloretin | n.d. | n.d. | n.d. | n.d. |
| Xanthoxumol | n.d. | n.d.–6.4 | n.d. | n.f |
| ***Lignans*** | | | | |
| Matairesinol | n.d. | n.d.–3.89 | n.d. | n.d. |
| ***Coumestan*** | | | | |
| Coumestrol | 7.8–143.4 | n.d.–1.74 | n.d.–0.627 | n.d.–0.33 |
| ***Phenylethanoil*** | | | | |
| Hydroxytyrosol | n.d–18.4 | n.d. | n.d. | n.d. |

n.d.: not detected; tr: tracers; μg/g: μg/g raw material

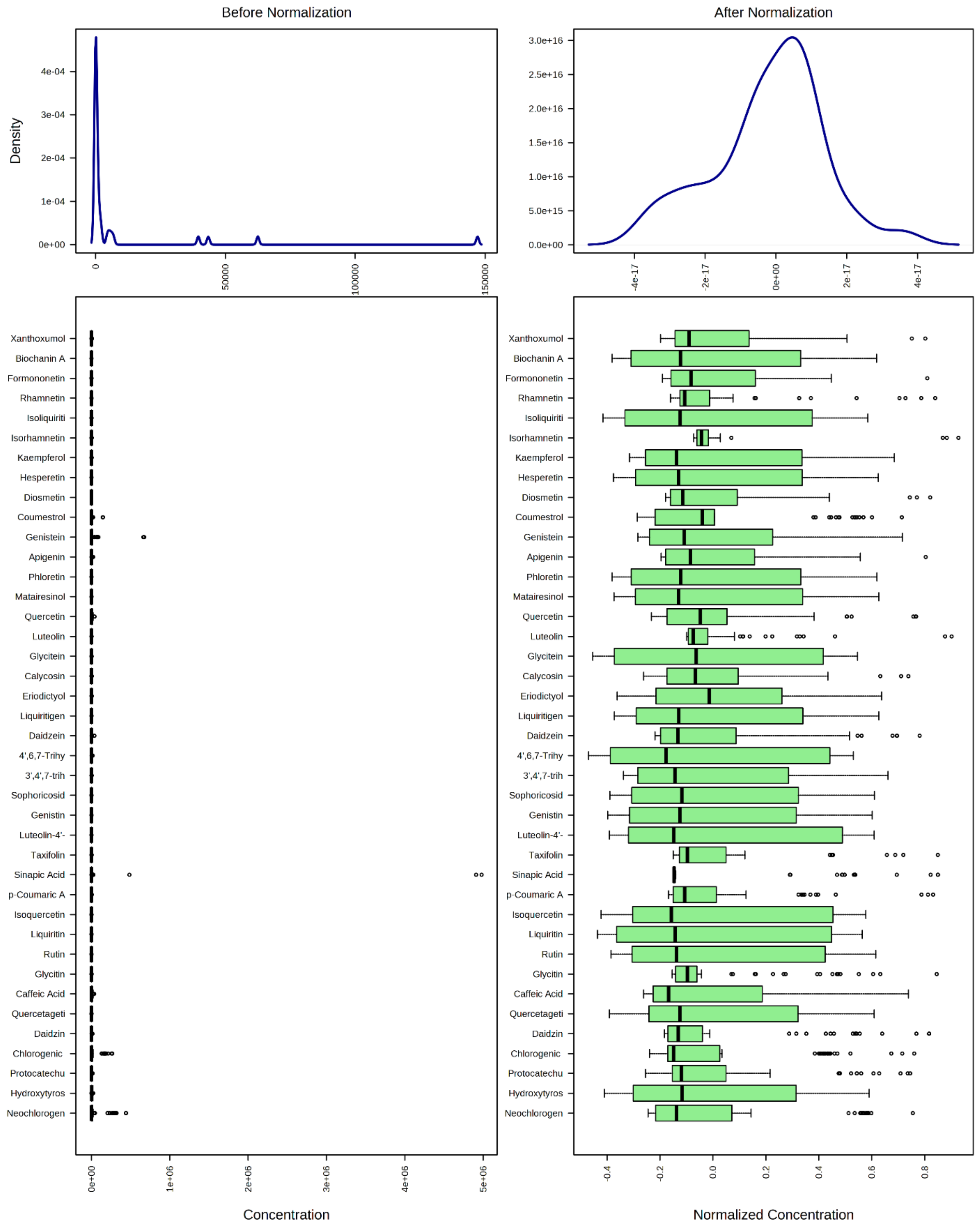


**Figure S1.** Concentration distributions before and after normalization based on the kernel density. Similarly, the bottom plots graphically summarize the concentrations of individual compounds.

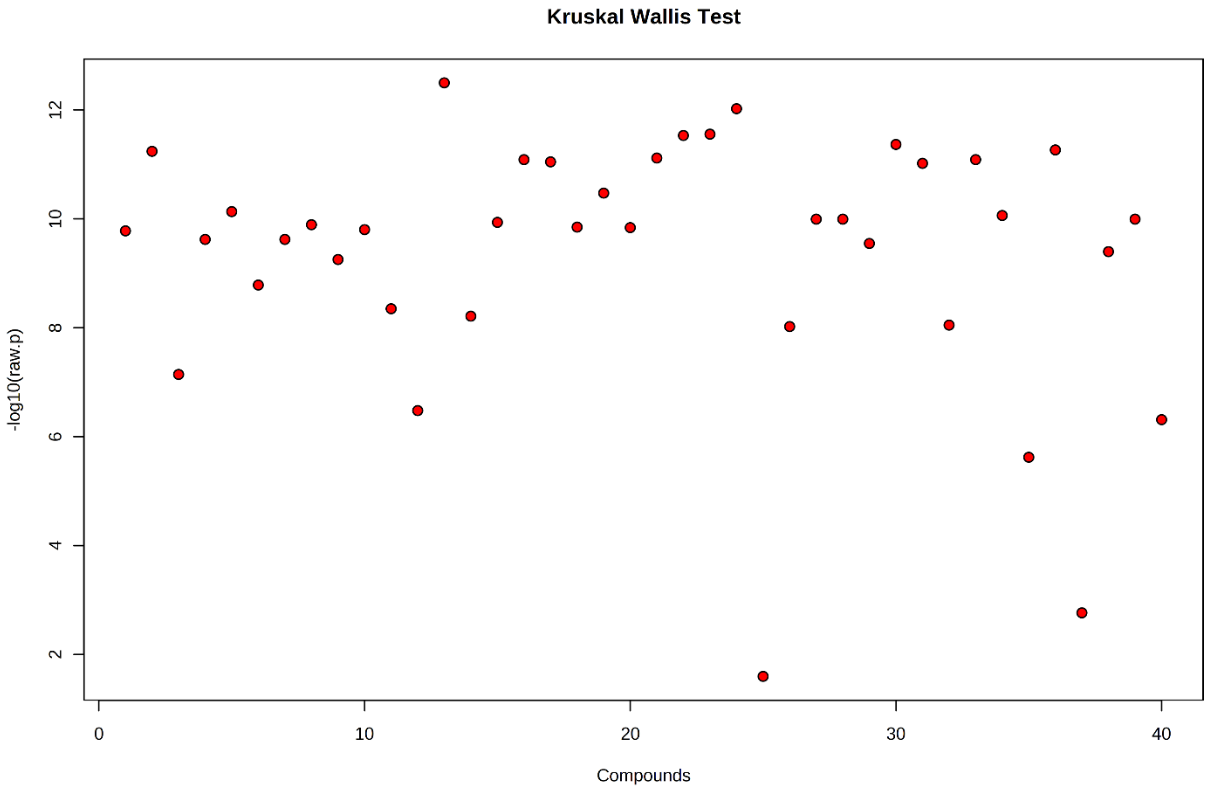


**Figure S2.** Results of the analysis of variance (ANOVA) of phytoestrogens, ranked by their P values (P<0.05).

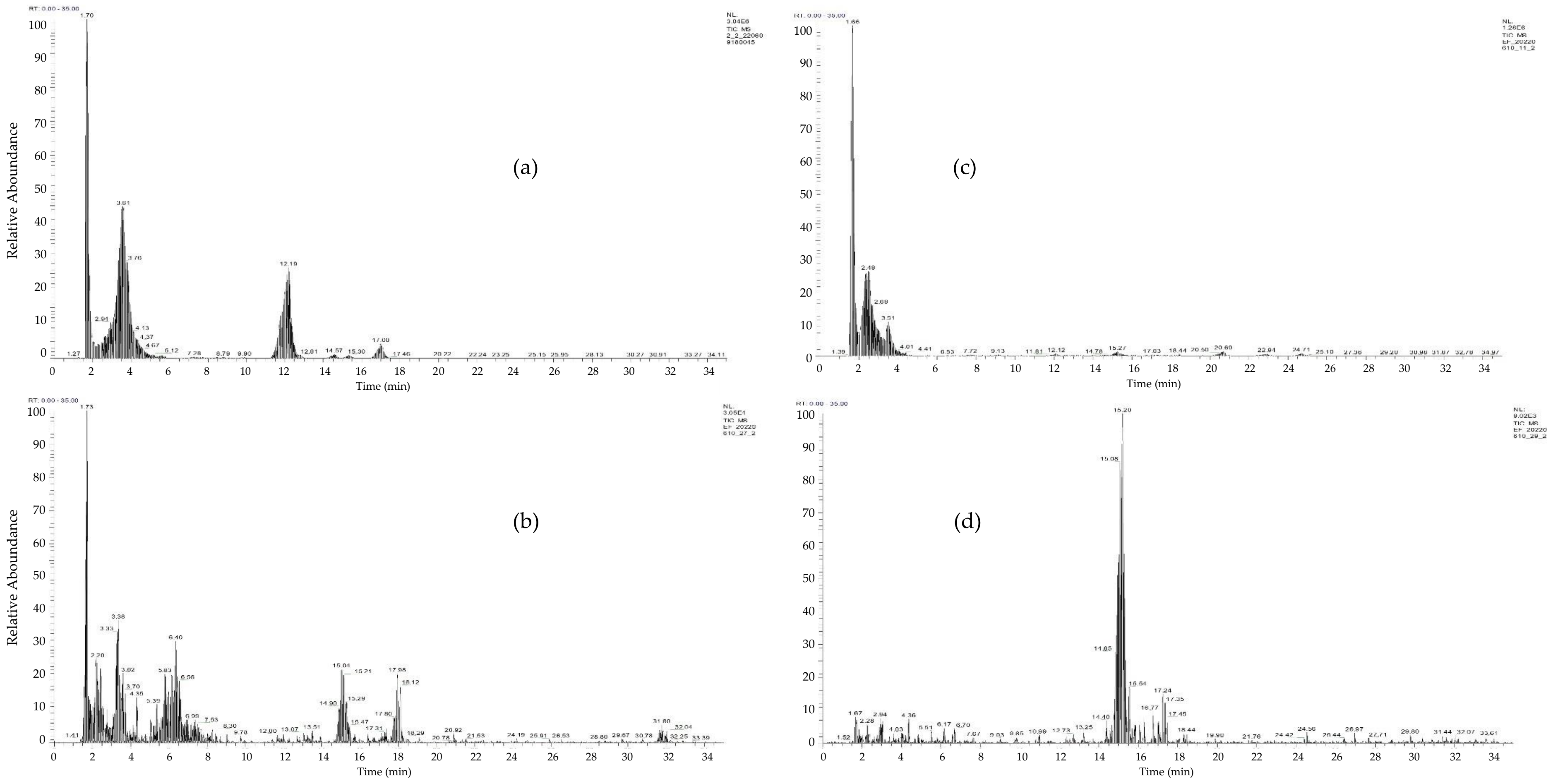


**Figure S3.** Chromatograms of phytoestrogens extracted from samples of: (a) soybean, (b) rapeseed, (c) sunflower and (d) wheat meals.
